# BRCA1/2 pathogenetic variant carriers and reproductive decisions: Gender differences and factors associated with the choice of preimplantation genetic diagnosis (PGD) and prenatal diagnosis (PND)

**DOI:** 10.1007/s10815-022-02523-y

**Published:** 2022-06-04

**Authors:** Lucia Lombardi, Carmen Trumello, Liborio Stuppia, Ivana Antonucci, Tânia Brandão, Alessandra Babore

**Affiliations:** 1 Department of Psychological, Health and Territorial Sciences, University “G. d’Annunzio” of Chieti-Pescara, Via Dei Vestini, 66100 Chieti, Italy; 2 Center for Advanced Studies and Technology-CAST, University “G. d’Annunzio” of Chieti-Pescara, Chieti, Italy; 3 CIP, Department of Psychology, Universidade Autónoma de Lisboa “Luís De Camões, Lisbon, Portugal; 4 CPUP, Center for Psychology, University of Porto, Porto, Portugal

**Keywords:** BRCA1/2, Decision-making, Human reproduction, Inheritance, Psychological adjustment, Review

## Abstract

**Purpose:**

To investigate the way carriers of a BRCA1/2 pathogenetic variant make their reproductive decisions and to examine the factors associated with the choice of preimplantation genetic diagnosis (PGD) and prenatal diagnosis (PND).

**Methods:**

We conducted a comprehensive literature search in PubMed, Scopus, and Web of Science in accordance with the Preferred Reporting Items for Systematic Reviews and Meta-Analyses (PRISMA) method.

**Results:**

A total of 16 articles published from 2000 to 2021 were included in this review. Data were overall collected from 3564 participants (86% females). Three important themes were identified across studies: changes in family planning, factors associated with family plans, and with acceptance or regret of PGD and PND.

**Conclusion:**

This review may contribute to the knowledge of the experience of those who have a BRCA1/2 mutation and want a child. These results may help genetic counselors and healthcare professionals that support people with a BRCA pathogenetic variant with reproductive issues.

## Introduction

For several years, 5/10% of cancer are estimated as hereditary and are accountable to mutations in susceptibility genes [1]. A mutation to BRCA 1 and 2 genes, which are considered important tumor suppressor genes, is a factor leading to hereditary breast and ovarian cancer syndrome (HBCD) [2]. Pathogenetic variants in BRCA1 and BRCA2 genes (chromosome 17q21 and chromosome 13q12, respectively) predispose female carriers to an increased risk of breast, ovarian, and pancreatic cancer as well as melanoma [3, 4] and male carriers to an increased risk of breast and prostatic cancer [5]. According to this, specific genetic test is available for the identification of high-risk persons [3]. Following the National Comprehensive Cancer Network guidelines [6], this category is mainly constituted by individuals with (1) a known BRCA1/2 pathogenetic variant within the family; (2) personal history of breast cancer (for example, early-onset diagnosis or additional breast cancer primary at any age); (3) a diagnosis of triple negative carcinoma; (4) personal history of ovarian carcinoma, or pancreatic cancer, or metastatic prostate cancer or male breast cancer; and (5) a family with many cancer cases (direct transmission) [6]. After detecting a BRCA1/2 pathogenetic variant, an active surveillance is proposed and, alternatively, preventive surgery (prophylactic bilateral salpingo-ophorectomy or mastectomy) or chemoprevention to reduce cancer risk [7]. This last option is particularly challenging in individuals who do not yet have children and who think they may have them in the future [8].

When the discovery of being a BRCA1/2 mutation carrier occurs within a couple who plan to have children, the couple is faced with challenging decisions regarding prophylactic treatment and future fertility decisions [9]. In fact, inheritance of these mutations is usually autosomal dominant, and carriers have a 50% risk of passing on the mutation to their offspring [10]. This situation increases not only the fear of developing a tumor but also the concern to pass the pathogenetic variant to the next generation [9]. Moreover, in families with several cases of cancer, the mutation diagnosis and the subsequent preventive measures may be experienced in a devastating way because parents are very frightened to transmit the mutation to their offspring, due to fear that they can live experiences similar to the past [11]. In these cases, reproductive decisions may be complex and difficult for couples with BRCA pathogenic variants, since the future child could present an increased risk of developing BRCA-related cancers [12].

Nowadays, couples with a BRCA1/2 pathogenetic variant who wants a child, or another child, can mainly have two reproductive options: to naturally conceive, without any type of diagnosis, and accept the risk of passing on the BRCA pathogenetic variant to the offspring or to choose techniques to prevent gene inheritance [13]. For this last option, two opportunities are available. The first one is the prenatal diagnosis (PND), made through chorionic villus sampling or amniocentesis. If a pathogenetic variant is found, parents have an important decision to take, that is whether to terminate or not the pregnancy [10]. The second option for couples is the genetic analysis of a preimplanted embryo (PGD), which is part of reproductive technology and is specifically used with an in vitro fertilization (IVF) or intracytoplasmic sperm injection (ICSI) cycle [9].

Therefore, the post-test genetic counseling for individuals in reproductive age includes not only bio-medical and psychosocial matters but also reproductive issues. People facing the possibility of passing on a pathogenetic variant to the offspring have to choose what to do in the event they want to expand the family.

This issue may arise in a psychological condition of increased level of distress, anxiety, and depression that previous literature has shown being associated with ﻿genetic test [14; for a review, see 15; 16].

Proceeding from these premises, the﻿ main aim of the current review was to examine the way carriers of a BRCA1/2 pathogenetic variant make their reproductive decisions and the factors associated with the choice of preimplantation genetic diagnosis (PGD) and PND. A further aim of this review was to analyze if gender may influence reproductive decision-making.

To pursue these objectives, we considered cross-sectional, longitudinal, and qualitative studies focusing on the impact of BRCA1/2 test results on carriers’ reproductive decision-making and their intentions and concerns about PGD and PND.

## Methods

### Systematic literature search

This systematic review was conducted in accordance with the Preferred Reporting Items for Systematic Reviews and Meta-Analyses (PRISMA) method [17, 18].

We conducted a comprehensive literature search in PubMed, Scopus, and Web of Science. We searched for studies published in the last 21 years (from 2000 to 2021), using the following keywords in combination: reproductive, BRCA, decision (reproductive AND BRCA AND decision). We performed also a manual search of reference lists in publications selected in order to identify all studies relevant for the present review.

### Eligibility criteria

Studies were included if they were written in the English language. We assessed the eligibility criteria according to the PICO(S) model [17]:

1. Participants: We investigated females and males with a BRCA1/2 pathogenetic variant and in reproductive age.
2. Intervention: We did not focus on a specific intervention.
3. Comparison: We compared studies on female carriers and male carriers.
4. Outcomes: We analyzed studies that investigated reproductive decision-making in couples with a BRCA1/2 pathogenetic variant and their attitudes about PGD and PND.
5. Study design: We included observational, quantitative, and qualitative studies.

Regarding exclusion criteria, articles were not considered eligible if they were not written in English, were specific on other hereditary syndromes, were literature reviews, letters to the editor, books, unpublished articles, doctoral theses, commentaries, abstracts of conferences, congresses, and case reports and if they were studies not reporting outcomes in line with the aim of the current review.

## Results

**Fig. 1:**
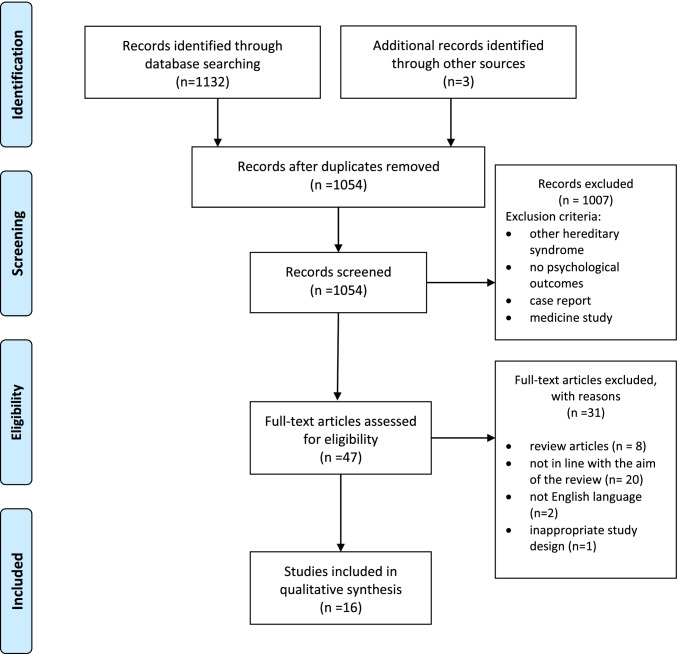
Flow chart of selection and inclusion process, following the PRISMA^1^ statement. *Note*: ^1^PRISMA, Preferred Reporting Items for Systematic Reviews and Meta-Analysis

The initial search conducted on July 22, 2021, returned a total of 1135 studies, screened for eligibility. Of these, 81 were identified as duplicates, and 1007 were excluded because they did not meet the eligibility criteria. The abstracts of 47 studies were double screened and evaluated by two independent authors and discrepancies were resolved by discussion. Among the examined studies, 8 were excluded because they were reviews, 20 studies were not in line with the aim of the review (dealing with such themes as communication of the pathogenetic variant in the family or psychosocial implications of living with BRCA pathogenetic variants), 2 were not written in the English language, and 1 had an inappropriate study design (case report). A total of 16 articles were included in this review (see flow chart in Fig. 1). These studies were published from 2000 to 2021 and the majority were conducted in the USA, but other countries were also represented (UK, Spain, the Netherlands, France, Israel). Regarding study design, half of the studies were quantitative and cross-sectional (*N* = 8; 50%), the remainder were qualitative studies (*N* = 7; 44%), and only one was a longitudinal study (6%).

Data were overall collected from 3564 participants (sample size of each study ranged from 20 to 1081 participants), and of these, 86% were females, and 14% were males. Regarding cancer diagnosis, 1144 (32%) of the whole sample had a history of a carcinoma diagnosis.

Of the included articles, six had a study population composed of only females, one article of only males, and the others included both genders.

### Tools and measures of the included studies

To assess how the BRCA status influenced reproductive decisions of participants, including their attitudes towards preimplantation genetic diagnosis and prenatal diagnosis for the BRCA pathogenetic variant, 7 (44%) of the included articles used interviews or semi-structured interviews [8, 11, 19–23], and 5 (32%) used ad hoc questionnaires [10, 24–27]. Two studies (12%) used ad hoc questionnaires in combination with validated questionnaires [9, 28], in specific, the Cancer Risk Perception (CRP) [29, 30] to assess the perception of the risk of run into a cancer diagnosis; the Perceived Health Status (PHS) [31] to assess the general health status; the Decision Regret Scale (DRS) [32] to assess decision-making processes; and the Satisfaction With Decision (SWD) scale [33] to assess satisfaction resulting from an important decision. Finally, two studies (12%) used semi-structured interviews in combination with validated questionnaires [34, 35], in this case, the Impact of Event Scale (IES) [36] to assess the distress associated with a specific event; the Hospital Anxiety and Depression Scale (HADS) [37] to assess anxiety and depression symptoms; and the Physical and Mental Health Short Form (SF-12) [38] to assess general health.

A summary of studies characteristics with the main results of each research is presented in Table 1.

**Table 1:** Characteristics of included studies

Author (year)	Country	Aim	Study design	Materials	Sample	Personal cancer history	Main results
Chan et al. (2017)	**USA**	Investigate how knowledge of BRCA status influences the decision to use PGD and PND.	Cross-sectional	Ad hoc questionnaire	Total sample = 1081 Women = 1081 Men = 0	History = 390 No history = 691	The presence of pathogenetic variant, personal history of cancer, and being single impacted on the decision to have a/another child.
Dekeuwer et al. (2013)	**France**	Investigate the way in which carriers of a mutation on the BRCA (1or 2) make their reproductive decisions.	Qualitative	Semi-structured interview	Total sample = 20 Women = 19 Men = 1	History = 12 No history = 8	Carriers are mainly concerned by the risk of transmitting “much more than a gene,” that is painful experiences.
Derks-Smeets et al. (2014)	**Netherlands**	Investigate how couples with a BRCA1/2mutation decide on PGD and PND.	Qualitative	Semi-structured interview	Total sample = 44 Women = 22 Men = 22	History = 2 No history = 42	Couples wanted to protect the future child from the BRCA pathogenetic variant. There were psychological motives to choose or reject PGD.
Donnely et al. (2013)	**UK**	Investigate how young women with a BRCA mutation approach reproductive decision-making.	Qualitative	Semi-structured interview	Total sample = 25 Women = 25 Men = 0	History = 6 No history = 19	Females with pathogenetic variant felt a sense of urgency to have a child.
Fortuny et al. (2009)	**Spain**	Explore the opinion about reproductive decisions among individuals undergoing BRCA1/2 testing.	Cross-sectional	Interviews HADS SF-12	Total sample = 77 Women = 67 Men = 10	History = 54 No history = 23	Cancer diagnosis was positively associated with PGD; older age and high educational level with PND.
Gietel-Habets et al. (2017)	**Netherlands**	Examine what is the attitude among couples with BRCA mutation towards reproductive options.	Cross-sectional	Ad hoc questionnaire	Total sample = 191 Women = 167 Men = 24	History = 40 No history = 151	Cancer diagnosis was positively associated with PGD; younger age and high educational level with PND.
Hurley et al. (2012)	**USA**	Explore the interesting of BRCA1/2 mutation carriers in learning about reproductive options.	Qualitative	Interviews	Total sample = 33 Women = 29 Men = 3	History = 0 No history = 33	The stress of genetic testing temporarily interfered with decision-making process.
Jiulian-Reynier et al. (2012)	**France**	Assess the impact of BRCA1/2 test results on carriers’ reproductive decision-making and their intentions about PGD and PND.	Cross-sectional	Ad hoc questionnaire, CRP, PHS	Total sample = 600 Women = 449 Men = 151	History = 0 No history = 600	Reproductive plans were accelerated in pathogenetic variant carriers because of the possibility to undergo preventive surgery. Males found PND more acceptable than females.
Menon et al. (2007)	**UK**	Explore opinion of BRCA carriers on preimplantation genetic diagnosis as a reproductive option.	Cross-sectional	Ad hoc questionnaire	Total sample = 54 Women = 54 Men = 0	History = 26 No history = 24	The majority of pathogenetic variant carriers are favorable that PGD is proposed, but most females would not personally consider it. Younger age, less religiosity, and cancer diagnosis were positively associated with PGD.
Mor et al. (2018)	**Israel**	Evaluate PGD uptake, decision satisfaction or regret, and predictors of uptake in BRCA mutation carriers.	Cross-sectional	Ad hoc questionnaire, DRS, SWD	Total sample = 70 Women = 70 Men = 0	History = 0 No history = 70	Previous infertility was the only significant predictor of PGD.
Ormondroyd et al. (2012)	**UK**	Explore reproductive decision-making, knowledge, and attitudes to reproductive options with women who received a positive BRCA test.	Qualitative	Semi-structured interviews	Total sample = 25 Women = 25 Men = 0	History = 6 No history = 19	PGD was considered acceptable because it would prevent transmission to future generations, but females had concerns about selecting embryos.
Quinn et al. (2010)	**UK**	Explore perceptions and attitudes towards PGD among males who either carry a BRCA mutation or have a partner or a first relative with BRCA mutation.	Cross-sectional	Ad hoc questionnaire	Total sample = 228 Women = 0 Men = 228	History = 7 No history = 221	Cancer diagnosis, age, religion, having children, and have a family member with pathogenetic variant were factors significantly associated with PGD.
Reumkens et al. (2018)	**Netherlands**	Investigate reproductive decision-making in couples with a high cancer risk mutation.	Qualitative	Semi-structured interviews	Total sample = 14 Women = 7 Men = 7	History = 0 No history = 14	Couples expressed a need for a complete explanation of the procedures and techniques used in PND and PGD in order to be helped to decide.
Smith et al. (2004)	**USA**	Test whether fertility intentions differed among persons who tested positive, tested negative, or did not know their genetic status for a mutation of the BRCA1 gene.	Longitudinal	Semi-structured interview, IES, Perceived risk of breast cancer	Total sample = 101 Women = 67 Men = 34 – Carriers = 25 Non carriers = 62 Unknown = 14	Not reported	Being a BRCA pathogenetic variant carrier was associated with lower intentions to have children (or additional children).
Vadaparampil et al. (2009)	**USA**	To assess sociodemographic, clinical, awareness, and attitudinal factors associated with acceptance of PGD among women at high risk of OC, BC.	Cross-sectional	Ad hoc questionnaire	Total sample = 962 Women = 962 Men = 0	History = 601 No history = 361	Significant predictors of PGD acceptance were the desire to have children, having had a prenatal genetic test, belief that PGD is acceptable, concerns about PGD, perceived benefits.
Werner-Lin et al. (2012)	**USA**	Investigate how BRCA1/2 mutation carriers understand genetic inheritance and consider a child’s inheritance.	Qualitative	Interviews	Total sample = 39 Women = 34 Men = 5	History = 0 No history = 39	Participants even with the genetic pathogenetic variant took the risk of transmitting it to their children and were more in favor of biological reproduction.

*PGD* preimplantation genetic diagnosis, *PND* prenatal diagnosis, *OC* ovarian cancer, *BC* breast cancer, *HADS* Hospital Anxiety and Depression Scale, *SF-12* Physical and Mental Health Short Form, *IES* Impact of Event Scale, *DRS* Decision Regret Scale, *SWD* Satisfaction With Decision scale, *CRP* Cancer Risk Perception, *PHS* Perceived Health Status

### Changes in family planning

As for the first aim of the current review, namely the way carriers of a BRCA1/2 pathogenetic variant make their reproductive decisions, ten of the considered articles explored this theme [8, 11, 19–21, 23, 24, 26, 28, 34]. Most of the included studies showed that the risk of passing on the pathogenetic variant may change the idea of starting a family or expanding the family [8, 11, 19, 20, 24, 28, 34]. In the study of Chan et al. [24], 17.2% of females decided not to have children because of the BRCA pathogenetic variant risk transmission to their offspring, and because of concerns that pregnancy might increase the risk of developing cancer. In the research of Dekeuwer et al. [11], the findings suggested that when carriers are planning to have a or another child, they are concerned by the risk of transmitting not only the pathogenetic variant but also painful experiences such as illness or cancer surveillance. Another study highlighted that couples wanted to protect the future child form the BRCA pathogenetic variant and this desire may influence the decision of expanding the family [8]. Donnelly et al. [19] underlined that carrier individuals were under pressure to have children as soon as possible, especially if preventive surgery was proposed, and this sense of urgency was particularly evident for females without children. According to this result, another study reported three major consequences of being a BRCA1/2 pathogenetic variant carrier: first, an acceleration of the respondents’ reproductive plans due to the possibility of risk-reducing salpingo-oophorectomy; second, the feeling of guilt about possible transmission of the gene pathogenetic variant to the present or future offspring; and third, the renunciation of the desire to have children [28].

In this scenario, the PGD and the PND are considered. PGD, compared to PND, is evaluated as more advantageous because it would prevent the transmission to future generations, but it produces more concerns due to the embryo selection [21]. For this reason, couples have expressed the need to be well informed about the procedures and techniques used in PGD and PND [22].

Hurley et al. [20] suggested that for some individuals, the stress of testing and communicating the pathogenetic variant temporarily interfered with information processing, making them not able to take a decision on family plans in a short time.

In the only longitudinal study considered in the current review, the authors highlighted that 2 years after genetic test confirming BRCA pathogenetic variant, females altered their family plan showing lower intentions to have additional children respect to not-carrier females. This difference was not significant between male carriers and not-carriers [34]. In fact, female pathogenetic variant carriers reported a reduction in fertility intentions [34]. In the study of Werner-Lin et al. [23], participants discussed how in the process of natural reproduction the genetic heritage is combined to produce something of unique and for this reason, individuals, even with the pathogenetic variant, took the risk of transmitting it to their children and were more inclined to biological reproduction.

In a study with only men, Quinn et al. [26] reported that some carriers strongly opposed to assisted reproductive technology, especially to PGD, because if it had been available to their parents, they would not have been born and for this reason, some carriers expressed doubts about moral justness of the decision they had to take [8], because, according to them, hereditary cancer syndrome was not a condition for which pregnancy termination was justified [21].

### Factors associated with family plans and acceptance or regret of PGD and PND

Consistent with the review aims, some factors associated with family planning and acceptance or regret of techniques to prevent gene inheritance, in specific PGD and PND, were analyzed. More specifically, 12 articles analyzed this issue [8–11, 20, 21, 24–28, 35]. Chan et al. [24] reported that females with a personal history of cancer were more likely to report that having the BRCA1/2 pathogenetic variant impacted their decision to have child than females without a cancer diagnosis, while women who were partnered were less likely to report that the BRCA status impacted their decision with respect to women without partners. Regarding PGD and PND, in this research, partner status and personal history of cancer were associated with the decision to have biological children but women who already had biological children were less likely to pursue fertility treatments.

In the research of Fortuny et al. [35], the authors reported that the diagnosis of cancer was positively associated with considering PGD. Also, Derks-Smeets et al. [8], Gietel-Habets et al. [10], and Ormondoryd et al. [21] underlined that the most important factor taken into account when couples make reproductive decision was the personal and familial experience with cancer. In contrast, in the research of Dekeuwer et al. [11], the risk of transmitting the pathogenetic variant was no less acceptable to females who had had breast or ovarian cancer than to those who had not.

In general, women who already had biological children were less likely to turn to assisted reproductive technology [24], because of the necessity of in vitro fertilization and low chance of pregnancy by IVF or PGD [8].

Derks-Smeets et al. [8] crafted a list of factors associated with both PGD acceptance and its regret. Among the most widely reported psychological motives to choose PGD, there were avoidance of feelings of guilt towards the child, preservation of control regarding pregnancy, faith in future medical developments, the BRCA1/2 pathogenetic variant elimination in family line, and avoidance of future pregnancy termination [8]. Among common psychological motives to refrain from PGD, there were loss of romance in couples, uncertainties during the assisted reproductive technology treatment, and high percentage of unsuccessful [8].

In other research, older age was the only variable significantly associated with considering PND [35]. All participants in this research reported that religious orientation did not influence their decision-making [35]. Also, in Mor et al. [9], the younger age and the religious beliefs were not associated to PGD, and a story of previous infertility was the only significant predictor of PGD. In contrast with these results, Menon et al. [25] highlighted that females who opted for PGD tended to be younger, less religious, more likely to have had breast cancer, and more concerned to transmit the gene pathogenetic variant to the offspring.

In general, it seemed that PGD was more acceptable than PND [28]. Julian-Reynier et al. [28] reported that PND acceptability was positively associated with lower educational levels and with male gender. Regarding the PGD, the decision did not depend on gender or age but was more present in those having no future childbearing plans and those having relatives with cancer [28]. In a wide research, the PGD acceptance was significantly associated with religion, the desire to have more children, and with previous prenatal genetic test [27].

### Gender differences in fertility intentions and in acceptance of PGD or PND

Only three studies investigated gender differences [20, 28, 34]. They found that PND acceptability was observed among males, but not among females, and that PGD was more acceptable than PND among women but not among men [28]. Male carriers were more predisposed towards reproduction techniques than female carriers, maybe because they do not face the number or degree of personal risks that female carriers face and their attitudes towards PGD may be shaped by the fact that they would not directly undergo the IVF procedure [20]. Smith and colleagues [34] reported that pathogenetic variant carriers were significantly less likely to report a desire for future children than not-carriers among females but not among males. Furthermore, a significantly lower interest in having additional children was reported only among female pathogenetic variant carriers but not among male carriers.

## Discussion

In this systematic review, we aimed to investigate the reproductive decision of BRCA1/2 pathogenetic variant carriers, and the factors associated with the choice of PGD or PND. We analyzed also gender differences in fertility intentions.

After a selection procedure following the PRISMA method [17], we considered 16 studies. The analysis of the selected articles showed rather diverse results. In general, the presence of a BRCA pathogenetic variant impacted on family planning and fertility intentions, but in a multifaceted way. Couples often experienced this reproductive decision-making process as difficult and decided not to have children because of BRCA transmission risk [24, 34]. In fact, fear and concern of transmitting the pathogenetic variant to the offspring are high [11], especially for the perception of passing “much more than a gene,” as the pathogenetic variant may imply frequent surveillance programs and the possibility of getting sick and suffering [11, 39]. In other cases, the couples experienced that they were under pressure to have children as soon as possible, accelerating their projects [19, 28], due to the possibility of preventive surgery; in fact, the higher the woman’s age, the higher the advice to undergo an ovariectomy [3]. Furthermore, female and male carriers were afraid of incurring into cancer diagnosis and having to perform treatments not compatible with fertility [24].

Regarding assisted reproductive technology as a method to avoid transmitting pathogenetic variant, the results are heterogeneous, and in some cases, the personal and familial experience with cancer is an important factor associated with acceptance or regret of PGD or PND [8, 10, 11, 21]. Probably, physical and mental suffering felt in cancer affects the decision process and the future parents do not want their child to live a similar experience [11]. Regarding age, research underlined that older age is associated with assisted reproductive technology use; in fact, in men and women with BRCA1/2 pathogenetic variant, the older the age, the greater the risk of developing cancer [3, 35]. This risk can likely accelerate the decision of having a child [28]. In contrast, another research highlights that females who opted for PGD tended to be younger, perhaps at the beginning of their family planning [25]. Two studies reported that the religion did not affect the decision to undergo assisted reproductive techniques [9, 35], while Menon et al. [25] highlighted that females who opted for PGD tended to be less religious, assuming that those who have a stronger religiosity ask more ethical questions about the medical procedures used.

The different results can be explained by the different geographical areas where the studies were conducted and by political, religious, and cultural factors that may influence the views of individuals and their reproductive decisions [40]. In fact, the three studies mentioned come from three different countries (Israel, Spain, UK). Furthermore, the heterogeneity of the studies depends also on the used instruments, which were almost all qualitative and not standardized.

Regarding gender, male carriers were more predisposed towards reproductive techniques than female carriers [28]. Their decision-making process was less influenced by mutation-bearing condition [20, 34]. In fact, male carriers reported a desire for future children like not-carriers [20]. Furthermore, it seemed that PND was more acceptable among men than among women [28]. An explanation of this finding could be that men are less involved at the physical level in assisted reproductive techniques, and this perhaps makes them more predisposed than women that instead experience all the medical procedures on their body.

## Study limitations and future implications

This review has some limitations. First, the considered studies refer to the last 20 years and the scientific and medical reproductive techniques in this long period have become more accurate and might have changed the patients’ perception, influencing acceptance or regret of PGD and PND. Furthermore, the majority of studies we analyzed included qualitative tools like semi-structured interviews and ad hoc questionnaires that were not standardized, and this might have affected the repeatability and the generalizability of research included in this review. Moreover, the sample size was unbalanced according to gender; in fact, females represented a great majority as compared with males. Another limitation regards the countries where studies were conducted: although there is a good representation, the majority of them are from America and UK. All these limitations lead us not to generalize our results.

However, the topic is complex and certainly needs further study. As we know, in fact, being a BRCA pathogenetic variant carrier has several consequences, both psychological and medical [15, 41]. As for the former, individuals may experience distress and a sense of precariousness and may show high levels of depression and anxiety [15, 16]. Furthermore, couples who want a/another child have to face the fear of developing a tumor and the concern to pass the pathogenetic variant to the next generation [9].

Regarding medical aspects, there are several theories on the effect of the BRCA pathogenetic variant on reproduction. Surprisingly, one of these suggests that BRCA carriers are significantly more fertile than others [42]. On the other hand, Oktay et al. [43] underlined that BRCA1/2 pathogenetic variants may unfavorably impact ovarian reserve. What is certain is that if individuals have already been diagnosed with cancer, drugs (such as chemotherapy) may interfere with the normal functioning of the gonads causing infertility [41, 44]. Furthermore, in these cases, it is recommended risk-reducing surgery also at younger age [6] . Another aspect to consider concerns ovarian stimulation, studies analyzing the relationship between infertility treatment and the incidence of ovarian cancer in women with BRCA pathogenetic variant, showed no differences between carriers and not-carriers [45] but there is no agreement on this issue in the scientific community [41, 45].

These are just some of the issues that couples with BRCA pathogenetic variants are facing. This review may contribute to the knowledge of the complex experience of those individuals who have a BRCA1/2 pathogenetic variant and want a child. These results may help genetic counselors and healthcare professionals that support people with BRCA pathogenetic variant. In fact, couples that are facing a BRCA1/2 pathogenetic variant may change their family planning influenced by the fear of cancer diagnosis and the risk of transmitting the pathogenetic variant to the offspring. Healthcare professionals have to better inform couples regarding risk of transmission and the possibility of assisted reproductive technologies, respecting the personal experience. It should be important that clinical psychologists participate to genetic counseling pre- and post-test, in order to offer psychological support during the decision process of couples. Furthermore, our data seem to underline the necessity to support women both in the decision-making process and eventually in the reproductive medical one. Regarding men, our data suggest that they should receive support in approaching the experience of their partners in order to better understand what they feel.

This review showed that further research is needed in this topic using more specific and standardized tools. Additionally, future studies should include more male samples and more countries.
